# Cognitive anosognosia and behavioral changes in probable Alzheimer's disease patients

**DOI:** 10.1590/S1980-57642013DN70200010

**Published:** 2013

**Authors:** Corina Satler, Carlos Tomaz

**Affiliations:** 1 PhD, Adjunct Professor, Faculty of Ceilandia, UnB, Brasilia DF, Brazil.; 2 PhD, Full Professor, Laboratory of Neurosciences and Behavior, Institute of Biology, UnB, Brasília DF, Brazil.

**Keywords:** awareness, anosognosia, insight, aging, Alzheimer's disease, dementia

## Abstract

Anosognosia, impairment insight and unawareness of deficits are used as equivalent terms in this study.

**OBJECTIVE:**

To investigate the relationship between the presence of anosognosia symptoms and cognitive domains, functional abilities, and neuropsychiatric symptoms in patients with probable Alzheimer's disease (pAD) and elderly controls (EC).

**METHODS:**

Twenty-one pAD (14 women) and twenty-two EC (16 women) were submitted to a neuropsychological battery of tests assessing global cognitive status, and specific cognitive functions: memory, executive and attention functions, verbal fluency and visuoconstructive abilities. Additionally, functional abilities (FAQ) and neuropsychiatric symptoms (NPI) were measured.

**RESULTS:**

The linear regression statistical test found general anosognosia to be associated with subjective memory complaints, age and Arithmetic-DRS in the EC group. On the other hand, cognitive and functional abilities scores (Arithmetic-DRS, IQCODE and FAQ) were the best predictors in pAD patients, particularly for behavioral awareness.

**CONCLUSION:**

These results indicated that different variables are associated with self-awareness for pAD patients and EC, but for both groups executive functions appear to play an important role, contributing particularly to awareness of behavioral changes.

## Abstract (pt)

Anosognosia, comprometimento do *insight* e ausência de reconhecimento de déficits são usados como termos equivalentes neste estudo.

**OBJETIVO:**

Investigar a relação entre a presença de sintomas de anosognosia e domínios cognitivos, habilidades funcionais, e sintomas neuropsiquiátricos em pacientes com provável doença de Alzheimer (pAD) e idosos controles.

**MÉTODOS:**

vinte e um pDA (14 mulheres) e vinte e dois EC (16 mulheres) foram submetidos a uma bateria neuropsicológica de testes avaliando o estado cognitivo global, e funções cognitivas específicas: memória, funções executivas e atencionais, fluência verbal e habilidades visoconstrutivas. Adicionalmente, habilidades funcionais (FAQ) e sintomas neuropsiquiátricos (NPI) foram avaliados.

**RESULTADOS:**

Teste estatístico de regressão linear indicou uma associação entre anosognosia e queixas subjetivas de memória, idade e aritmética-DRS no grupo controle. Por outro lado, aritmetica-DRS, IQCODE e FAQ (habilidades cognitivas e funcionais) foram melhores predictores nos pacientes com pDA, especificamente para consciência das alterações comportamentais.

**CONCLUSÃO:**

Os resultados indicaram que diferentes variáveis estão associadas com auto-percepção nos pacientes com pDA e controles, porém para ambos os grupos parece que o funcionamento executivo tem um papel importante contribuindo principalmente na percepção das alterações comportamentais.

## INTRODUCTION

The term "anosognosia" was originally coined to describe the lack of insight into hemiplegia following right hemisphere stroke.^1^ It is important to note that, whereas insight was once considered relevant only to hemiplegia, it is now recognized as applicable to cognitive and behavioral domains affected by neurological disorders other than stroke, such as dementia.^1^ In this article, "anosognosia" and the equivalent terms "impaired insight" and "unawareness of deficits" are used in this strict sense.

Alzheimer's disease (AD) is the most common form of dementia, and is a pathology where we can observe anosognosia symptoms^2,3^ that can occur in up to 81% of individuals with AD.^4^ However, there is considerable inter-person variability in the presentation of anosognosia in AD.^5^

Among AD patients, studies have demonstrated that anosognosia is common in early stages of the disease, and have suggested that impairment of insight increases with severity of the dementia.^6^

Previous studies seeking to identify the association between awareness of cognitive deficits in AD have shown mixed results. In short, the mechanisms underlying insight in AD remain unclear. For example, some authors^2,5,7^ have found a significant correlation between unawareness of deficits and global cognitive function, using the Mini-Mental State Examination (MMSE) or Clinical Dementia Rating (CDR). In contrast, other researchers have not found evidence of this association.^8^

In addition, some studies have also found relationships between psychiatric symptoms and anosognosia in AD.^2,7,9^

More specifically, it has been demonstrated that there is a difference between the absence of perception of cognitive deficits and of behavioral disorders. Studies have described that AD patients show impaired insight for memory and cognitive deficits, and daily living activities deficits, which constitute core symptoms of AD, but also for a range of other possible symptoms including different cognitive deficits, and psychiatric symptoms such as depression, anxiety, or irritability.^9^ However, there is no clear evidence that anosognosia for cognitive deficits and behavioral alterations in AD differs with functional and neuropsychological performance.

One example of the awareness of memory deficits in dementia is the presence of subjective memory complaints that reflect preservation of the ability to recognize and evaluate one's own deficits. Global cognitive function would be a pre-requisite for appropriate judgment. Thus, Leicht, Berwing, and Gertz^3^ described that while AD patients tended to overestimate their cognitive abilities, in older adults without cognitive impairment there is an underestimation of ability.

It has been reported that memory complaints are a feature that seem to be common among middle-aged and older adults without dementia,^10,11^ and that the prevalence of memory complaints tends to be higher as age increases. For example, in the Maastricht Aging Study, the prevalence of forgetfulness among volunteers was 41% in the group aged 55 to 65 years and 52% in the 70 to 85 year-old age group.^10^

Jorm and colleagues,^12^ in a community sample study, described that memory complaints were associated with poorer cognitive test performance, more prominent mood symptoms, personality characteristics and poor physical health. In other words, cognitive performance was not only contributed to by memory complaints.

In the present study, we attempted to examine the presence of lack of awareness of specific deficits (intellectual functioning and behavioral changes) in AD patients, and explore a possible relationship with cognitive and neuropsychiatric profiles and functional impairments.

In order to examine the presence of anosognosia in elderly adults without cognitive impairment, we also explored the relationship between awareness of deficits and cognitive performance.

## METHODS

**Participants.** We carried out a case control study in a convenience sample of 43 participants, 21 of whom were patients diagnosed with probable AD (pAD) and 22 healthy elderly controls (EC). pAD patients were recruited from a larger pool of participants at the Geriatric Medical Center, University Hospital of Brasilia, Brasilia, Brazil, and were examined by a social worker, a neuropsychologist, and a geriatrician. Healthy controls were recruited from the community or were spouses of the patients and were examined with a comprehensive neuropsychological and neuropsychiatry evaluation, and had no history of either neurological or psychiatric disorders, including traumatic brain injury or substance abuse.

All AD patients met the clinical diagnostic criteria of AD described by the National Institute of Neurological and Communicative Diseases and Stroke-Alzheimer's Disease and Related Disorders Association.^13^ The diagnoses by the study geriatricians were based on the neuropsychological, medical, and laboratory findings. The severity of AD ranged from mild to moderate (scores 1 or 2) according to the Clinical Dementia Rating scale.^14^ All patients exhibited a 1 to 4-year history of progressive cognitive impairment, predominantly affecting memory, which was confirmed by their caregiver using the short version of the IQCODE (Informant Questionnaire on Cognitive Decline in the Elderly),^15^ but showed normal consciousness and lived with their families and required no special care.

All patients and control volunteers had normal or corrected-to-normal vision and hearing. The neuropsychiatric Inventory was applied to all subjects.^16^ If any evidence of behavior disturbance or significant depression was noted after the interview, the subject was excluded.

**Instruments and procedures.** After confirming the diagnosis for those patients that met the inclusion criteria of the study and selection of participants for the control group, the neuropsychological evaluation was performed on each individual in both groups by the same investigator. All participants were evaluated using the neuropsychological tests during two sessions on separate days (with an interval of 7 days), and tests were always applied in the same order.

*Neuropsychological assessments.* As part of the initial assessment, standardized neuropsychological tests were used to assess different cognitive functions.

*Global Cognition Score.* A Brazilian version of the MMSE,^17^ and the Mattis Dementia Rating Scale (DRS), was used.^18^ In addition, the Vocabulary subtest (the Wechsler Adult Intelligence Scale - WAIS-III)^19^ was used as a measure of premorbid intelligence.

*Memory.* The Digit Span Forward (DRS)^18^ and Corsi's Block-Tapping Test^20^ assessed short-term memory (verbal and visual memory, respectively).

The Auditory Verbal Learning Test (RAVLT)^21^ was used for assessing the learning curve, strength of memory after interference task, and pattern of learning (serial positioning effects), as well as for measuring recognition memory.

The 15-item version of the Boston Naming Test (BNT) in Consortium to Establish a Registry for Alzheimer's Disease^22^ measured the ability to name pictures of objects (semantic memory), and the Rey-Osterrieth Complex Figure (ROCF) - recall^20^ assessed nonverbal memory.

*Executive and Attention Functions.* Executive function and attention measures were assessed using the following tests: the Clock Drawing Test (CDT) - to command,^23^ Wisconsin Card Sort Test (WCST) - modified version,^24^ the Weigl Test,^20^ the Stroop Color-Word Test (SCWT)^20^ and the 5-Point Test (FPT).^20^ These tests evaluated planning, set shifting, selective attention, speed of visuomotor coordination, and divided attention.

Additionally, all participants performed simple arithmetic tasks (addition, multiplication, subtraction, division) using the Arithmetic subtest - DRS^18^ and mental manipulation of well learned sequences was assessed by the Mental Control subtest - DRS.^18^

*Verbal Fluency.* Verbal fluency tests such as Animal naming and words beginning with letters "FAS"^20^ were used to evaluate phonological and lexical retrieval in oral modality.

*Visuoconstructive Abilities.* The CDT - copy^23^ and the ROCF - copy^20^ were used to assess visuospatial skills. In the CDT, subjects are asked to draw a clock and to set the time at 45 past two. This task is used as a test of constructional abilities, but also requires some level of executive control.

*Measure of functional abilities.* These values were obtained using a questionnaire instrument filled in by an informal caregiver, such as a family member. The Pfeffer Functional Activities Questionnaire - FAQ^25^ was used to identify the level of functionality of the subjects required in the instrumental activities of their daily routine.

Additionally, subjective memory complaints were analyzed using a self-report memory questionnaire - MAC-Q.^26^

*Neuropsychiatric symptoms.* The Neuropsychiatric Inventory (NPI) was used to measure psychopathology in the participants; this measure was also developed specifically for use with dementia. The NPI rates the severity and frequency of 12 neuropsychiatric disturbances that are common in dementia: delusions, hallucinations, agitation, dysphoria, anxiety, apathy, irritability, euphoria, disinhibition, aberrant motor behavior, night-time behavior disturbances, and appetite and eating abnormalities together with the related caregivers' distress.

Depressive mood was assessed using the Cornell Scale for Depression in Dementia (CSDD).^27^

As with the measures of functional abilities, the same informal caregiver was interviewed by the assessor who filled in the scales.

*Anosognosia symptoms assessment.* The Brazilian version of the Anosognosia Questionnaire-Dementia (AQ-D)^28^ was used in this study. Originally, this questionnaire was devised to examine the effects of clinical variables, such as cognitive functions and the presence of neuropsychiatric symptoms, on these domains of anosognosia in patients with AD.

The AQ-D is a 42-item questionnaire collecting responses from both patients and their caregivers about the current level of impairment of patients. The AQ-D consists of questions divided into two sections: The first section assesses intellectual functioning, while the second section examines changes in interests and personality. Each answer is rated as never (0 points), sometimes (1 point), usually (2 points), or always present (3 points). Thus, higher scores indicate more severe impairments.

Form A is answered by the patient alone (with clarifications by the examiner if needed), whereas form B (a similar questionnaire written in the third person) is answered by the patient's caregiver blind to the patient's answers in form A.

The final score is the subtraction of scores in forms A from scores in form B. Thus, positive scores indicate that the caregiver rated the patient as more impaired than the patient's own evaluation (the patient was less aware of his or her cognitive and emotional deficits).

All participants were tested individually in a room with normal interior lighting.

**Ethical aspects.** Written, informed consent in accordance with the ethical guidelines for research with human subjects (196/96 CNS/MS resolution) was obtained from all participants and their caregivers (where appropriate). The study was approved by the Human Subject Committee of the Faculty of Medicine, University of Brasilia.

**Statistical methods.** A descriptive analysis (means and standard deviation) was undertaken for the socio-demographic variables of the sample. In order to evaluate the neuropsychological data, t tests for independent samples were performed for each test.

Pearson's correlation coefficient was employed for correlation of scores between AD-Q and the other instruments. Linear regression models were used to evaluate the association between the anosognosia scores (total score, behavioral changes score and intellectual functioning score) and the cognitive processes, the level of functionality of the subjects and neuropsychiatric symptoms.

The significance level was set at p<0.05(two-tailed) for all tests.

## RESULTS

Socio-demographic characteristics of patients and controls are depicted in Table 1.

**Table 1:** Demographic characteristics in AD patients and elderly controls

	AD (n=21)	EC (n=22)
Age (years)	78.62±6.66	71.00±6.85
Range	65-88	65-84
Gender (M:F)	7:14	8:16
Education level (years)	6.81±4.08	12.32±4.42
Range	15-04	12-04
FAQ**	19.19±7.27	0.32±1.04
NPI total score*	17.33±12.09	6.23±7.45
CSDD total score*	10.33±6.91	5.50±3.88
IQCODE score	3.90±0.60	2.88±0.52
MAC-Q score	26.10±5.77	25.45±6.61
AQ-D cognitive changes score**	25.48±16.15	5.45±7.72
AQ-D behavioral changes score*	8.81±10.31	3.23±5.74
AQ-D total score**	34.76± 24.77	8.95±12.25

AD: Alzheimer's disease; EC: Elderly controls; M:F: Male:Female; FAQ: Functional Activities Questionnaire; NPI: Neuropsychiatric Inventory; CSDD: Cornell Scale for Depression in Dementia; IQCODE: Informant Questionnaire on Cognitive Decline in Elderly; MAC-Q: Memory Complaint Questionnaire; AQ-D: Anosognosia Questionnaire for Dementia. Values expressed as means±SD or Percentage.

* p<0.05;

** p<0.01 (Student's t-test).

Mean age was 74 years (pAD patients: 78.62±6.66 years; EC: 71.00±6.85 years), school education was 9.62 years on average (pAD patients: 6.81±4.08 years; EC: 12.32±4.42 years). Groups did not differ significantly with respect to these variables [age (p=0.864), education (p=0.808)]; however, pAD patients were older and had less formal education than EC.

Group performance was similar for the IQCODE test (p=0.583) and the MAC-Q (p=0.429). Notably, the scores for both groups on the MAC-Q were higher than the cut-off score (<25), (pAD patients: 26.10±5.77; EC: 25.45±6.61) suggesting the presence of subjective memory complaints. Additionally, it is also important to note that the pAD patients showed higher scores on the CSDD compared to EC (p=0.009).

*Neuropsychological assessment.* Neuropsychological results are listed in Table 2 and revealed a significant main difference among EC and pAD patients which reached statistical significance on most tests except the 5-Point Test-perseverations (p=0.713). In general, t tests showed that pAD patients performed significantly worse than EC.

**Table 2:** Test performance of AD patients and elderly controls.

Measure	AD	EC
**Global cognition score**		
MMSE, n/ 30**	18.00±4.29	28.64±1.39
DRS, n/ 144**	113.33±8.45	139.27±3.70
Vocabularysubtest (WAIS-R)**	32.48±10.14	49.27±8.33
**Short-term memory**		
Digit Span Forward (DRS)**	4.95±1.32	6.64±1.13
Corsi' Block-TappingTest Forward*	4.81±1.91	6.50±1.94
**Episodic recall**		
RAVLT		
Trial 1	2.00±1.61	5.45±1.43
Trial 2	2.90±2.09	7.77±1.82
Trial 3	3.38±2.24	9.59±2.42
Trial 4	3.76±2.11	9.72±1.93
Trial 5	4.04±2.65	11.00±1.87
Delayed Recall	0.95±2.15	8.36±2.40
Delayed Recognition	-9.38±14.05	10.86±3.50
**Semantic recall**		
BNT, n/ 15**	12.48±2.13	15±0.00
**Nonverbal recall**		
ROCF - Recall**	3.00±3.13	18.00±6.10
**Working Memory**		
Digit Span Backward (DRS)**	2.62±1.39	4.41±1.18
Corsi' Block Test Backward**	2.81±1.53	5.23±1.27
**Calculation ability **		
Arithmeticsubtest (DRS)**	3.33±0.61	7.50±0.85
**Executive and Attention functions **		
CDT - To command**	7.38±2.51	9.82±0.50
WCST - Categories**	1.57±1.59	4.05±1.67
WCST - Total errors**	20.14±12.28	7.27±5.74
FPT - Unique designs**	5.19±4.39	18.41±7.71
FPT - Perseverations, %	2.43±2.78	2.14±2.37
StroopTest - Interference condition,n/32**	13.95±8.05	27.27±5.60
Mental Control subtest (DRS)**	5.29±2.77	8.68±0.64
**Verbal Fluency**		
Letters (FAS)**	16.86±10.05	36.45±10.63
Categories (animal)	5.57±2.74	17.77±4.33
**Visuoconstructive abilities**		
CDT - copy**	4.57±2.95	9.55±0.85
ROCF - copy**	19.12±12.13	33.5±2.63

AD: Alzheimer's disease; EC: Elderly controls; MMSE: Mini-Mental State Examination; DRS: Mattis Dementia Rating Scale; RAVLT: Rey's auditory verbal learning test, trial 1 - trial 5, delayed recall score is the number of words reproduced from the 15 words given, recognition range 0 - 30 (score of 15 is chance and 30 indicates complete recognition);50 BNT: Boston Naming test; ROCF: Rey-Osterrieth Complex Figure; CDT: Clock Drawing test; WCST: Wisconsin Card Sorting test; FPT: Five-Point test. Values expressed as means±SD.

* p < 0.05;

** p < 0.01 (Student's t-test). Higher test scores indicate better performances.

*AQ-D Intellectual functioning.* The performance of the AD group on two cognitive tests (DRS - total and FPT - unique designs) was inversely associated with the degree of anosognosia (r(2) = 0.748; p<0.001), which explained 51.1% of the variance (DRS - total score: 42.1% and FPT - unique designs: 9%). Additionally, two other variables contributed positively to the increase of anosognosia symptoms: Letter FAS (15.8%) and NPI (7.9%). For the EC group, MAC-Q score and age variables were inversely associated with degree of anosognosia (R(2)=0.38; p= 0.004), explaining 38% of the variance (MAC-Q: 26.2% and age: 12.2%) (Table 3).

**Table 3:** Significant variables associated with anosognosia symptoms.

		b	EP	T	p-value	R2	F	gl	p-value
**AQ-D Intellectual Functioning**									
EC	Constant	56.53	16.52	3.42	0.003	0.38	7.55	2.19	0.004
	MAC-Q	-0.76	0.21	-3.67	0.002				
	Age	-0.45	0.20	-2.22	0.038				
AD Patients	Constant	200.80	28.88	6.96	<0.001	0.75	15.85	4.16	<0.001
	DRS total score	-1.66	0.28	-6.02	<0.001				
	FPT - Unique designs	-1.65	0.45	-3.65	0.002				
	Letters (FAS)	0.86	0.24	3.61	0.002				
	NPI	0.38	0.15	2.51	0.023				
**AQ-D Behavior changes**									
EC	Constant	-55.62	15.87	-3.50	0.002	0.4	8.10	2.19	0.003
	Arithmetic subtest (DRS)	3.82	1.12	3.39	0.003				
	Mental Control subtest (DRS)	3.48	1.50	2.32	0.032				
AD Patients	Constant	-63.35	8.80	-7.20	<0.001	0.83	21.31	5.15	<0.001
	Arithmetic subtest (DRS)	2.20	0.42	5.29	<0.001				
	IQCODE	12.33	2.01	6.11	<0.001				
	FAQ	0.72	0.15	4.86	<0.001				
	RAVLT - delayed recall	-1.29	0.52	-2.47	0.026				
	Stroop - interference cond.	0.30	0.14	2.15	0.048				
**AQ-D Total score**									
EC	Constant	42.11	32.22	1.31	0.208	0.572	10.36	3.18	<0.001
	Arithmetic subtest (DRS)	5.33	2.18	2.45	0.025				
	MAC-Q	-1.05	0.29	-3.63	0.002				
	Age	-0.65	0.28	-2.38	0.029				
AD Patients	Constant	-41.33	32.20	-1.28	0.217	0.821	23.96	4.16	<0.001
	Arithmetic subtest (DRS)	4.10	0.97	4.25	0,001				
	IQCODE	17.55	4.90	3.58	0.002				
	FAQ	2.05	0.34	6.04	0,000				
	BNT	-3.65	1.37	-2.65	0.017				

AD: Alzheimer's disease; EC: Elderly controls; AQ-D: Anosognosia Questionnaire in Dementia; MAC-Q: Memory Complaint Questionnaire; DRS: Mattis Dementia Rating Scale; FPT: Five-Point test; NPI: Neuropsychiatric Inventory; IQCODE: Informant Questionnaire on Cognitive Decline in Elderly; FAQ: Functional Activities Questionnaire; RAVLT: Rey's auditory verbal learning test; BNT: Boston Naming test.

*AQ-D behavior changes.* In the AD group, cognitive and functional abilities were associated with degree of anosognosia (R(2)=0.835; p<0.001), which explained 83.5% of the variance (IQCODE test: 44.1%, FAQ: 19%, Arithmetic subtest: 19%, Stroop test - interference condition: 3.7%, and RAVLT- delayed recall: 5.2% being inversely proportional). For the EC group, performance on two cognitive tasks (Arithmetic subtest - DRS and Mental control subtest - DRS) was associated with the presence of anosognosia symptoms focused on behavior changes (R(2)=0.403; p=0.003). Both cognitive variables explained 40.3% of the variance (Arithmetic subtest - DRS: 27.3% and Mental control subtest - DRS: 13%) (Table 3). *AQ-D total score.* In the AD group, cognitive and functional abilities scores were associated with degree of anosognosia (R(2)=0.821; p<0.001), which explained 80% of the variance (IQCODE test: 38.8%, FAQ: 23.4%, Arithmetic subtest - DRS: 13.6% and BNT: 6.3%; where the latter variable was inversely proportional to the presence of anosognosia symptoms) (Table 3).

For the EC group, three variables were associated with the presence of anosognosia symptoms (R(2)= 0.572; p<0.001), which explained 57.2% of the variance: Arithmetic subtest - DRS (29.9%), MAC-Q (16.8%) and age (10.5%). Two of these variables, MAC-Q and age, were inversely proportional to anosognosia symptoms (Table 3).

## DISCUSSION

The aim of this study was to explore a possible relationship between the presence of anosognosia symptoms and cognitive domains, functional abilities, and neuropsychiatric symptoms in pAD patients and normal elderly controls.

The main finding of this study was a significant difference between groups regarding the presence of anosognosia symptoms. Results showed that pAD patients were less accurate in evaluating the level of their awareness of cognitive and behavioral changes than healthy older adults, as evidenced by a larger discrepancy between self-and informant ratings on the AQ-D in pAD patients compared to EC. These findings corroborate a long line of research that suggests the presence of anosognosia for cognitive deficits and behavioral changes is a common and early feature of AD.^7^

**Ad patients and anosognosia.** Examining awareness of intellectual functioning, results showed a strong association with global cognitive functioning (DRS - total score). A similar result has been described by Sato and colleagues^29^ using the same anosognosia questionnaire (AD-Q), and the MMSE as a screening test for dementia. Thus, our findings showing that preserved awareness was associated with more intact cognitive functioning supports previous research.^2,6,7,30^

Furthermore, the association between verbal and design fluency tasks (FAS test and FPT respectively) with intellectual awareness suggests that pAD patients who have poor executive functioning might show more anosognosia for intellectual deficits, as described by other studies,^31^ including an association with specific brain alterations such as those involving frontal lobe regions.^32^

Loss of awareness, as well as psychosis and apathy, has been associated with neuropsychological changes in right frontal and related subcortical structures.^4^ Similarly, our findings showed a modest association between awareness for cognitive deficits and the NPI suggesting that pAD patients with behavioral disturbances failed to appreciate the degree of their cognitive impairment and often under-estimated it.

Levels of unawareness of behavioral changes were associated with higher scores on the IQCODE. These findings suggested that pAD patients with impaired insight were described by their caregivers and family members as having more cognitive changes over the last 10 years.

We also found an association between behavioral awareness and functional abilities (FAQ), a finding also described by previous studies.^29,33^ This association suggests that patients with a higher level of independence have more insight about self-care, in contrast to patients that exhibit functional impairment.

pAD patients with functional impairment are unable to perform some complex tasks, such as cooking, managing their finances, shopping, using the telephone, and taking medications. Considering that these activities require a greater variety of cognitive abilities, usually involving higher order cognitive processes,^34^ several studies have described that cognitive impairment is strongly associated with alterations in IADL.^35,36^ Along the same lines, Barberger-Gateau and colleagues^37^ found a relationship between performance on neuropsychological tests and measures of IADL and high risk for developing dementia.

In addition, our findings showed that results on neuropsychological tests were associated with loss of awareness of behavioral problems, namely, math skills such as numerical and calculation tasks (Arithmetic subtest - DRS), attention and response inhibition (Stroop test), and delayed recall of word-list (RAVLT - delayed recall).

In the area of arithmetic, there are differences in the strategies people use to solve an arithmetic problem. For example, the same person might solve a simple subtraction problem by counting and solve another by retrieving the answer from memory.^38^ Arithmetic fact retrieval is very likely to involve lexical processes, in that arithmetic facts appear to be represented in the same memory system that supports world retrieval, called "semantic memory". Thus, according to the literature, multiplication sums such as 3 x 4 = 12 are assumed to be stored in long-term memory.^39^

In addition, several studies have shown the role of the prefrontal cortex in mathematical performance, particularly in actual mathematical calculation or reasoning.^40^ In school-age children and adolescents, mathematical skills are associated with executive functions ^41^, and Bull and Scerif^42^ have also noted the role of specific executive functions, such as working memory and inhibition in mathematical proficiency.

In the Stroop test, the incongruent condition consists of naming the color of ink in which a color name is printed when the color is incongruent with the name. This condition elicits the Stroop effect that implies a significant slowing of performance. The response tendency or task set of reading the word, through life-long experience with reading is initially activated more strongly than the novel response tendency or task set naming the color. A number of studies have shown a deficit in Stroop test performance by patients with pAD, and evidence suggests that this deficit is due to a breakdown in inhibitory processes that occurs early in the course of the disease.^43^ These results are in line with those obtained by other studies suggesting that pathology for frontal brain regions is typically associated with executive dysfunction and also with poor behavioral insight.^44^

It is also found that episodic memory impairments (word recall - RAVLT) are associated with an increase in behavioral awareness. Difficulty with the acquisition of new information is generally the first and most salient symptom to emerge in patients with pAD. Studies describe the loss of episodic memory as the core feature of the dementing processes,^45^ and these observations are consistent with findings that brain structures known to be critical to episodic recall, such as the hippocampus and neighboring regions, show pathological alterations in pAD patients.^46,47^ In addition, there is evidence that the delayed recall score of word-list learning tasks, such as RAVLT, is the best neuropsychological parameter for discriminating pAD patients from healthy controls.^48^

Previous studies have described an association between anosognosia and behavioral changes in pAD patients, and have correlated the lack of awareness of their deficits with disease progress, and marked cognitive and functional impairment.^2,5,30^

Despite the relative lack of awareness of the severity of everyday cognitive declines and behavioral changes (total score in AD-Q), our findings showed that the same main factors that specifically contributed to behavioral awareness (IQCODE, FAQ, and Arithmetic subtest DRS), were also associated with general anosognosia, suggesting that behavioral changes tend to be a better predictor of anosognosia in AD patients.

Regarding a possible association between anosognosia and symptoms of depressed mood in AD patients, our results showed that the lack of awareness of their deficits is not influenced by the presence of depressed symptoms, as described by another studies.^2,4,28,30^

**Elderly controls and anosognosia.** In the evaluation of elderly controls, there was a negative association between awareness for intellectual functioning and subjective memory complaints. Consequently, those elderly that reported subjective memory complaints seemed able to pay attention to their own cognitive functioning and exhibit preservation in self-awareness.

These findings are in agreement with previous studies describing that memory complaints are frequent in healthy elderly population.^11,49^ However, given performance levels of these participants on neuropsychological tests were according to their age and educational level, it can be said that participants' complaints of memory are not an accurate reflection of real memory disturbance. Thus, it seems the over-estimation in healthy Brazilian elders may be related to cultural factors.

Regarding awareness of behavioral changes, our findings showed that math skills (Arithmetic subtest - DRS) and executive control (Mental control subtest - DRS) were associated with awareness of behavioral problems, suggesting executive functions are involved in this process. Although elderly controls did not exhibit high anosognosia scores, an interesting pattern was observed similar to that observed in pAD patients.

When analyzing total scores, the variables that contributed to intellectual functioning and behavioral changes were associated with general anosognosia. The findings differ in this regard from the general pattern observed in pAD patients where mainly behavioral changes contributed to general anosognosia.

Considered as a whole, we found that different variables were associated with self-awareness for pAD patients and elderly controls, but for both groups it seems that executive functions play an important role in contributing to awareness of behavioral changes.

Our findings in this report are subject to at least four limitations. First, data on the educational level of relatives or proxies were not collected, and this point should be addressed in further studies since this may have influenced the IQCODE results. Second, the diagnosis of anosognosia was based on cut-off scores on a severity rating scale rather than on valid diagnostic criteria for anosognosia. Third, this study investigated awareness deficits in pAD patients considering discrepancies between informant and self-reports in order to establish anosognosia. Although this approach can be valid when shown to correspond to objective performance, it is potentially influenced by informants' personal characteristics and prone to inaccuracy, as reported by Zanetti and colleagues.^50^ Finally, the small sample size is a shortcoming that limits this study, and any conclusion should be interpreted with caution. Therefore, further studies, including larger samples of controls and pAD patients, as well as other neuropsychological tests, are necessary to assess the association with awareness of deficits in the early stage of AD.
